# Ultrasound-Guided Transvaginal Aspiration and Sclerotherapy for Uterine Cystic Adenomyosis: Case Report and Literature Review

**DOI:** 10.3389/fmed.2022.764523

**Published:** 2022-03-03

**Authors:** Xinxin Zhao, Ye Yang

**Affiliations:** ^1^Department of Hospice, Sheng Jing Hospital of China Medical University, Shenyang, China; ^2^Department of Ultrasound, Sheng Jing Hospital of China Medical University, Shenyang, China

**Keywords:** adenomyosis, aspiration, cystic adenomyosis, sclerotherapy, ultrasound, uterine tumor

## Abstract

**Background:**

Uterine cystic adenomyosis is a very rare type of adenomyosis which can be easily misdiagnosed in clinical practice. In the past, cases have been mostly treated with surgical resection of the uterine lesion.

**Case Presentation:**

We report the case of a 25-year-old woman who presented with severe dysmenorrhea for more than 1 year. Physical examination showed that the uterus was enlarged. The transvaginal ultrasound showed a cystic mass of about 5.0 × 3.6 × 3.6 cm in the posterior myometrium, with dense echo spots and no blood flow signal in the cystic part. Magnetic resonance imaging (MRI) indicated hemorrhages within the cystic mass, suggesting the possibility of uterine cystic adenomyosis. The lower abdominal pain and severe dysmenorrhea were not alleviated after a 6-month trial of oral contraceptives. Subsequently, she underwent ultrasound-guided transvaginal aspiration and sclerotherapy for uterine cystic adenomyosis. Approximately 90 mL of chocolate-colored fluid was aspirated from the mass and 20 mL of lauromacrogol was injected in the cyst. The reduction rates of the mass 3 and 12 months after the procedure were 92.01 and 99.10%, respectively. Her dysmenorrhea completely resolved. One and half year after the operation, she had a successful pregnancy and gave birth to a healthy baby through vagina.

**Conclusion:**

The rare entity of uterine cystic adenomyosis can be treated safely and effectively by ultrasound-guided transvaginal aspiration and sclerotherapy.

## Introduction

Adenomyosis is a common gynecological condition characterized by the abnormal presence of endometrial glands and stroma within the myometrium (1). Menstrual bleeding within the ectopic endometrial tissue can lead to cystic foci. These cystic foci are usually small, with the largest diameter usually being <5 mm (2). However, in very rare cases, the cystic foci acquire diameters >1 cm thus constituting uterine cystic adenomyosis (3). The endometrial-like tissue in uterine cystic adenomyosis sheds with the menstrual cycle, leading to hemorrhagic infarction of the adjacent smooth muscle, and accumulation of bloody fluid that increases the volume of the cyst. Enlarged cysts can cause symptoms such as menorrhagia, infertility, pelvic pain, and severe dysmenorrhea (3). These symptoms are often not effectively controlled pharmacologically and need to be treated by surgical removal of the uterine lesion.

In this report, we present a rare case of uterine cystic adenomyosis that was treated by ultrasound-guided transvaginal aspiration and sclerotherapy. The diagnosis and treatment strategies for uterine cystic adenomyosis are discussed by summarizing and analyzing relevant literature over the past 30 years.

## Case Presentation

A 25-year-old woman presented to our hospital with “severe dysmenorrhea for more than 1 year” as the main complaint. She had a regular menstrual cycle of 28 days. During her menstrual period, she experienced lower abdominal pain and occasional back pain. In the past year, her dysmenorrhea had gradually deteriorated. Routine blood tests were normal, and serum tumor markers were not tested. She denied having a history of malignancy, endometriosis, genetic and psychosocial diseases, or prior surgeries.

**Figure 1:**
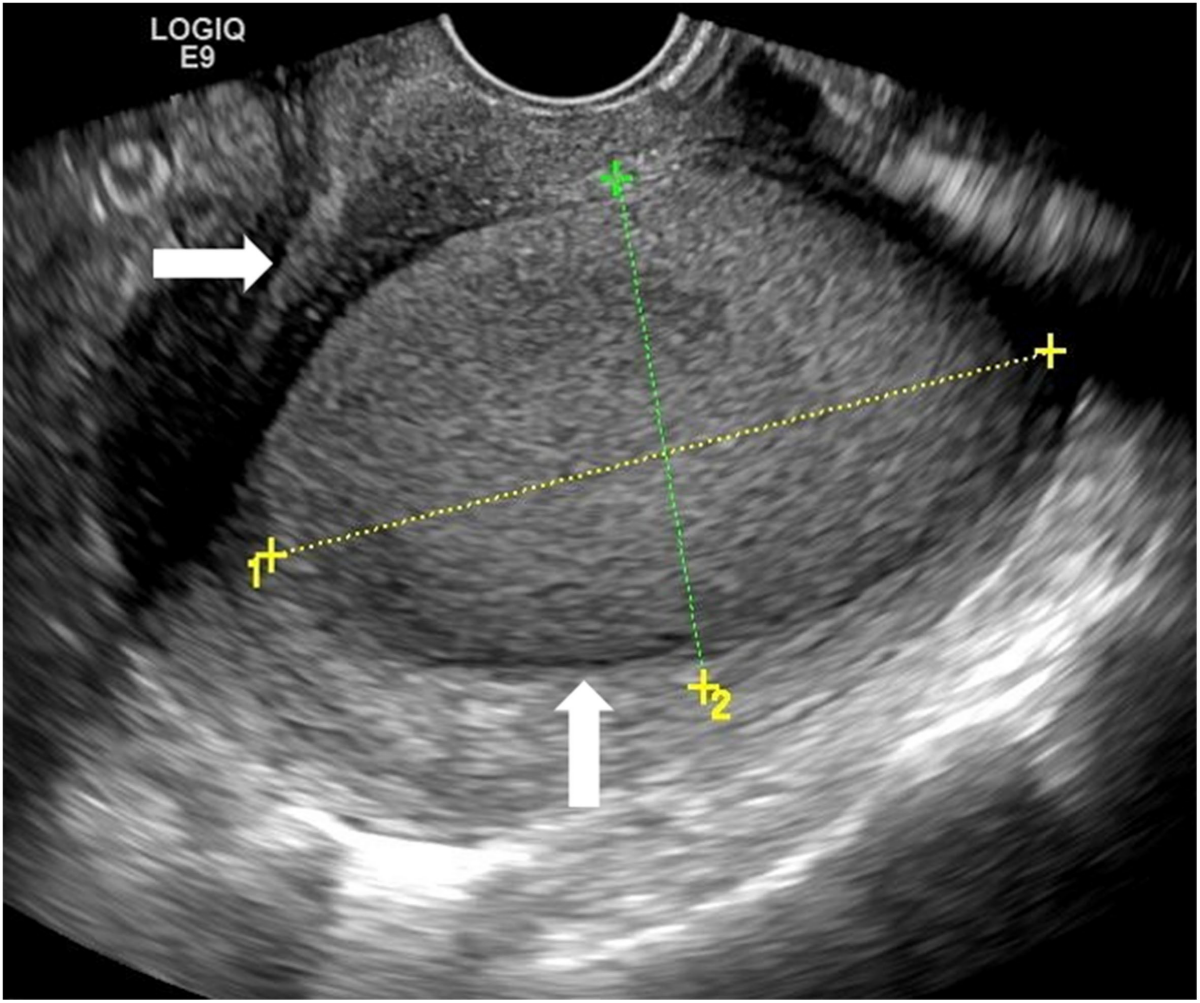
Transvaginal ultrasound showed a cystic mass (vertical arrow) in the posterior myometrium, accompanied by dense echo spots. The uterine cavity (horizontal arrow) did not communicate with the mass.

**Figure 2:**
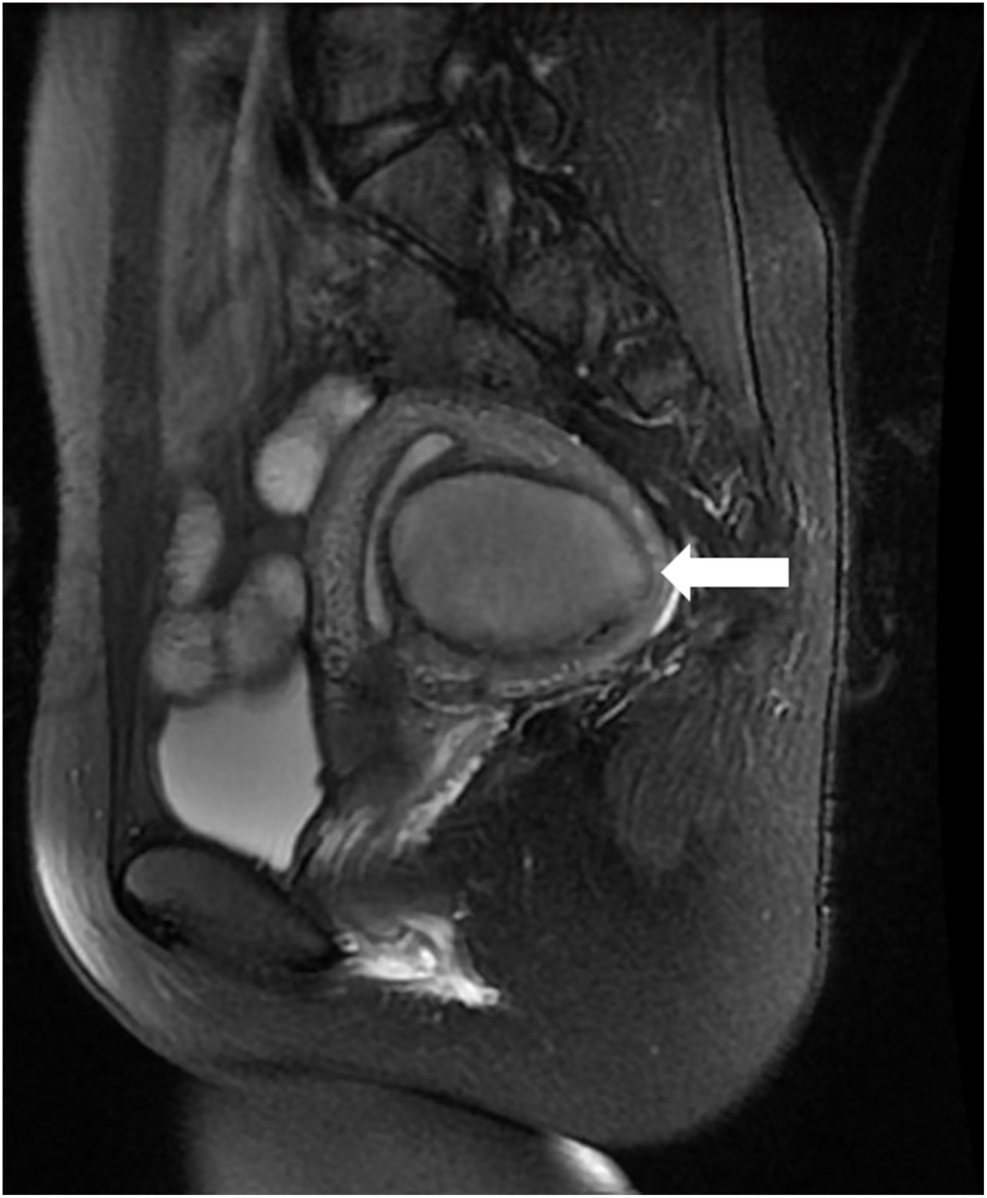
The mass (horizontal arrow) showed hyperintense signal on T-2 weighted Magnetic resonance imaging (MRI) image. It was protruding outward compressing the endometrium but not communicating with the uterine cavity.

Physical examination showed that the uterus was enlarged and slightly hard on palpation, and the posterior wall protruded outward locally. Transabdominal ultrasound in another hospital suggested a mass, not otherwise specified, in the uterine wall; therefore, a transvaginal ultrasound examination was scheduled. Transvaginal ultrasound showed that the uterus was enlarged, and a cystic mass, well-circumscribed and ellipsoid, ~5.0 × 3.6 × 3.6 cm in size was identified in the posterior uterine myometrium (Figure 1). Dense echo spots were observed in the liquid. Color Doppler flow detected weak blood flow in the cystic wall, but no blood flow within the cyst. The myometrium around the mass was compressed and thinned. The shape of the uterine cavity was normal, and the cystic mass was well separated from the uterine cavity. Bilateral ovaries were normal without space-occupying lesions. We considered the uterine cystic mass to be the result of prior hemorrhage. We, therefore, proceeded with magnetic resonance imaging (MRI) to further clarify the nature of the mass. MRI showed that the oval-like cystic mass was located in the posterior wall of the uterus, with a regular shape and smooth and clear boundaries (Figure 2). The cystic part showed a hyperintense signal on both T1-weighted and T-2 weighted images, with a few flocculent equal signals. There was no obvious enhancement in the mass on enhanced scanning. MRI also indicated hemorrhages within the cystic mass, suggesting the possibility of uterine cystic adenomyosis. Combined with the patient's history of severe dysmenorrhea, the characteristics of transvaginal ultrasound and MRI, a diagnosis of uterine cystic adenomyosis was established.

The patient was started on continuous oral contraceptive (OC) pills for 6 months, without, however, improvement of her lower abdominal pain and severe dysmenorrhea. Six months later, ultrasound examination showed that the mass had increased to 7.5 × 5.4 × 4.8 cm. At that point surgical treatment was recommended. Considering that the patient was a young woman who might wish to become pregnant in the future, several uterine-preserving treatment methods were discussed with her in detail, among whom she opted for the method of ultrasound-guided transvaginal aspiration and sclerotherapy and provided informed consent. The research protocol was approved by the medical ethics committee of our hospital and participants gave written informed consent, according to CARE guidelines and in compliance with the Declaration of Helsinki principles. Transvaginal sclerotherapy was performed for uterine cystic adenomyosis under ultrasound guidance on the 10th day of the menstrual cycle. The patient was placed in the lithotomy position, and 2% lidocaine was administered locally. A percutaneous transhepatic cholangiography (PTC) needle (18G) was inserted into the cystic mass, and the position of the needle tip was verified by ultrasound. Approximately 90 mL of chocolate-colored fluid was aspirated from the mass, and part of the fluid was sent for pathological examination. The cyst cavity was repeatedly flushed with normal saline until the color of the aspiration fluid was clear, and all the fluid from the cyst was drained. While flushing the cyst cavity, we observed that there was no leakage of normal saline in the uterus, thus indicating lack of communication between the cyst and the uterine cavity. Lauromacrogol injection (10 ml, 0.1 g) was used for sclerotherapy treatment. Two branches of Lauromacrogol injections (20 ml) were injected into the cyst through a PTC needle and finally retained. The procedure lasted 45 min and the patient was observed for about 4 hours after the operation. During and after the injection, the patient had no discomfort and no adverse events.

Cytological examination of the aspirate revealed hemosiderin-laden macrophages, without tumor cells, epithelial or mesenchymal components. The chocolate-like appearance and cytological characteristics of the aspirated contents confirmed the diagnosis of uterine cystic adenomyosis.

**Figure 3:**
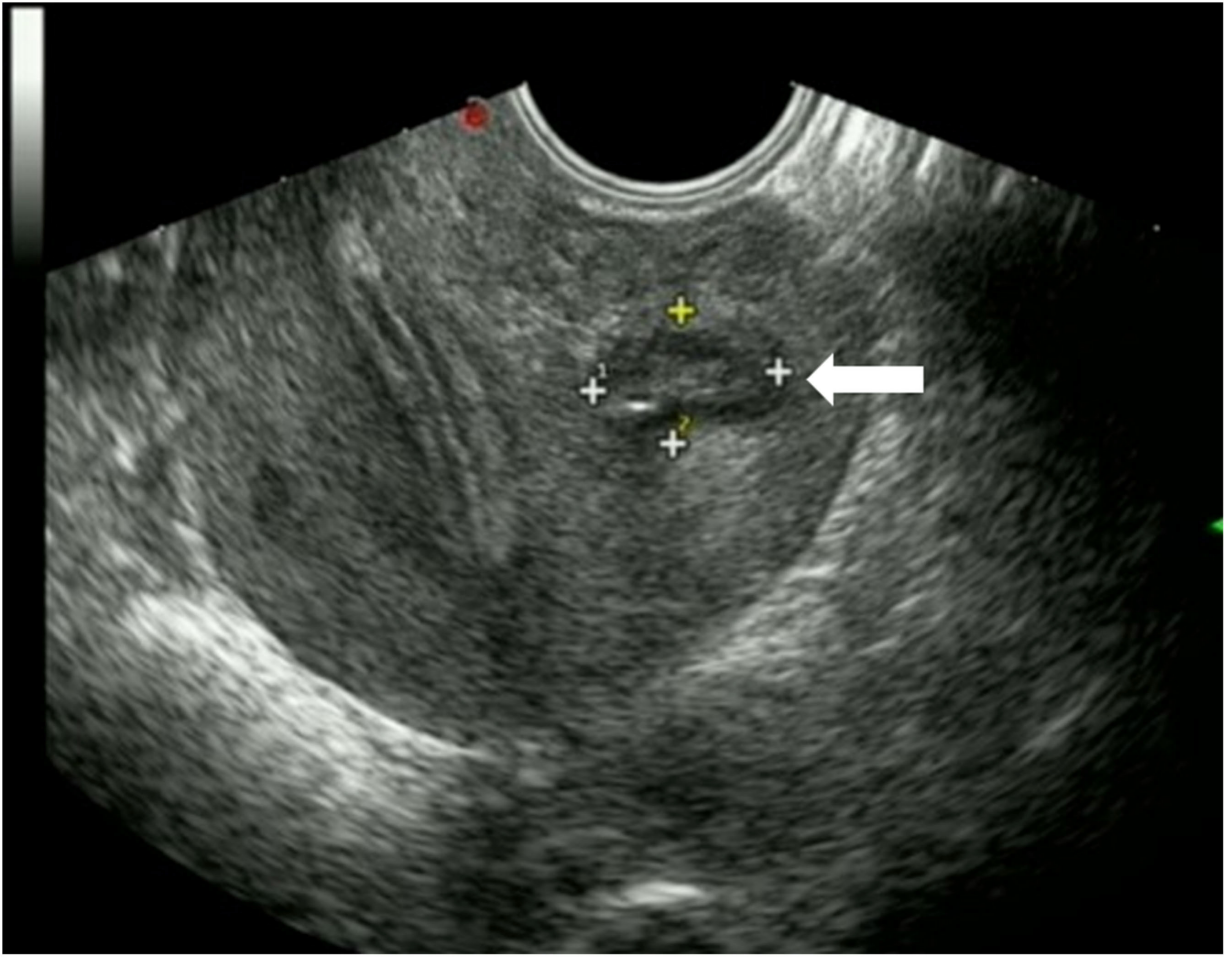
Transvaginal ultrasound showed that the mass (horizontal arrow) was significantly decreased 1 year after operation, with moderate echo and no fluid collection.

Three months after the procedure, ultrasound examination showed that the mass had decreased to 3.4 × 2.2 × 2.0 cm, corresponding to a volume reduction rate of 92.01%. A small fluid collection was still visible. One year after the operation, the mass had decreased to 1.4 × 1.2 × 1.0 cm, corresponding to a volume reduction rate of 99.10%, and it was moderately echogenic, with no fluid present, appearing similar to a scar-like structure (Figure 3). The thickness and echo of the myometrium around the mass were normal, and the thickness of the endometrium was within the normal range. After the operation, the patient had regular menstruation and no symptoms of dysmenorrhea or abdominal pain. Based on the improvement of the patient's clinical symptoms and imaging manifestations, the operator believed that the effect of this treatment was very significant. One and half year after the operation, the patient became pregnant and successfully delivered a healthy baby through vagina at 40 weeks of pregnancy in our hospital. We recommended that the patient undergo MRI of the uterus before and after delivery, but she refused. Ultrasonography showed no cyst formation in the uterine wall. The patient never experienced delivery complications and any sclerotherapy-related adverse events, she was very satisfied with the treatment.

## Discussion

In 1908, Cullen first described uterine cystic lesions filled with chocolate-colored fluid (4). As a special type of endometriosis, the incidence of uterine cystic adenomyosis is low, and related literature reports in the past 30 years are shown in Table 1 (5–46). Terms often used in the literature include juvenile cystic adenomyosis (JCA), cystic myometrial lesions, juvenile adenomyotic cysts, uterine cystic adenomyosis, adenomyotic cyst of the uterus, adenomyotic cyst, intramyometrial cystic adenomyosis, or intrauterine cystic adenomyosis. Compared with adenomyosis, the age of onset of this cystic form is younger, with the average age in the literature being 29.5 years, and 60.71% (34/56) of the patients being younger than 30 years. The most common symptoms are severe dysmenorrhea and pelvic pain, while some patients also experience irregular menstruation. Dysmenorrhea in women with uterine cystic adenomyoma can be explained by the progressive increase in cyst size resulting from repeated intra-cystic bleeding during menstruation.

**Table 1:** Case reports of uterine cystic adenomyosis.

**References**	**Age (years)**	**Involvement**	**Imaging**	**Number of lesions**	**Maximum size (cm)**	**Menstrual cycles**	**Symptom**	**SerumCA125 (<35U/mL)**	**Preoperative diagnosis**	**Diagnosis method**	**Treatment**	**Outcome**	**Surgery history**
Ejeckam GC et al. (5)	35	Posterior uterine wall	US	1	20 × 15.5 × 12.6	Regular	Pelvic and back pains, menorrhagia	Not mentioned	Multiocular cyst	Microscopic evaluation	Hysterectomy	Not mentioned	Not mentioned
Iribarne C et al. (6)	26	Posterior aspect of the uterus and the fundus	US	1	3.5 × 3.1	Not mentioned	Primary sterility	Not mentioned	Intramyometrial cyst of adenomyotic origin	Cytology	Laparotomy cystectomy	Not mentioned	Not mentioned
Tamura M et al. (7)	16	Left uterine myometrium	US, MRI, laparoscopy	1	3	Regular	Dysmenorrhea	Not mentioned	Cyst-like structure	Microscopic examination	Laparotomy cyst removal	Recovery	Not mentioned
Kataoka ML et al. (8)	28	Myometrium	US, MRI	1	3.0 × 2.5 × 2.2	Not mentioned	Severe dysmenorrhea	37	Myometrium lesion	Histopathology	Laparotomy cyst removal	Not mentioned	Not mentioned
Kataoka ML et al. (8)	44	Subserosal of left uterine fundus	MRI	1	4 × 4 × 3	Not mentioned	Dysmenorrhea and left lower abdominal pain	51	Myometrium lesion	Histopathology	Laparotomy cyst removal	Not mentioned	Not mentioned
Kataoka ML et al. (8)	46	Posterior to the uterus	US, MRI	1	12 × 9 × 8.5	Not mentioned	Lower abdominal distension and lumbago	69	Degenerated subserosal leiomyoma	Histopathology	Laparotomy cyst removal	Not mentioned	Not mentioned
Nabeshima H et al. (9)	19	Right uterine wall	US, MRI	1	3	Not mentioned	Severe dysmenorrhea	40.9	Cystic-like mass	Microscopic examination	Laparotomy cyst removal	Recovery	Not mentioned
Imaoka I et al. (10)	41	Left uterine myometrium	US, MRI	1	8 × 8 × 6	Irregular	Dysmenorrhoea and hypermenorrhoea	673	Left malignant ovarian tumor associated with endometriosis	Histopathology	Hysterectomy and bilateral salpingo-oophorectomy	Recovery	Not mentioned
Koga K et al. (11)	37	Uterine wall	MRI	1	17 × 11 × 8	Not mentioned	Abdominal cramps and menorrhagia	Not mentioned	Cystic adenomyosis	Microscopic examination	Hysterectomy	Not mentioned	Myomectomy twice, curettage twice for evacuation of a hydatidiform mole
Ryo E et al. (12)	21	Right uterine myometrium	US, MRI	1	3	Not mentioned	Dysmenorrhea	40.4	Adenomyotic cyst	Microscopic examination	Radiofrequency ablation	Recovery	Not mentioned
Fisseha S et al. (13)	13	Left uterine myometrium	US, MRI	1	2.9 × 2.2	Not mentioned	Pelvic pain and episodic vaginal bleeding	Not mentioned	Cystic adenomyosis	Clinical manifestations	Oral contraceptive	Clinically stable and asymptomatic	Not mentioned
Kamio M et al. (14)	23	Left anterior uterine myometrium	US, MRI	1	3 × 3	Regular	Severe dysmenorrhea, menorrhagia, and abdominal cramps	2.5	Isolated adenomyotic cyst	Histopathology	Laparotomy cyst removal	Recovery	Not mentioned
Takeda A et al. (15)	20	Right anterior uterine myometrium	US, MRI	1	3	Not mentioned	Severe dysmenorrhea	25	Cystic adenomyosis	Histopathology	Laparotomy cyst removal	Recovery	No
Takeda A et al. (15)	20	Left anterior uterine myometrium	US, MRI	1	2.6	Not mentioned	Severe dysmenorrhea	40.5	Cystic adenomyoma	Histopathology	Laparotomy cyst removal	Recovery	No
Yamashiro T et al. (16)	39	Intrauterine and expanding into the subserosa	MRI	1	14 × 12 × 10	Not mentioned	Abdominal mass	44	Uterine sarcoma with hemorrhage	Histopathology	Hysterectomy	Not mentioned	Not mentioned
Ho ML et al. (17)	16	Right uterine myometrium	US, CT, MRI, laparoscopy	1	Not mentioned	Not mentioned	Cyclic pelvic pain	Not mentioned	Not mentioned	Histopathology	Laparotomy	Recovery	Not mentioned
Ohta Y et al. (18)	54	Uterine fundus	US, CT, MRI	1	11 × 11 × 10	Not mentioned	Hypermenorrhea	Not mentioned	Not mentioned	Histopathology	Hysterectomy	Liver metastasis	Not mentioned
Dogan E et al. (19)	19	Left anterior wall of uterus	US, MRI, laparoscopy	1	4	Not mentioned	Severe dysmenorrhea	Normal	Intramyometrial mass	Histopathology	Laparotomy cyst removal	Recovery	Not mentioned
Akar ME et al. (20)	15	Right uterine wall	CT, US, laparoscopy	1	4.8 × 3.4 × 3.0	Not mentioned	Severe dysmenorrhea	Not mentioned	Cystic adenomyoma	Histopathology	Robot-assisted laparoscopic cyst removal	Not mentioned	Not mentioned
Kriplani A et al. (21)	16	Right side of posterior uterine wall	Not mentioned	1	4 × 4	Not mentioned	Severe secondary dysmenorrhea	Not mentioned	Degenerated myoma, bicornate uterus with noncomunicating horn	Histopathology	Laparoscopic removal of cyst	Recovery	Not mentioned
Kriplani A et al. (21)	18	Right side of uterine wall	Not mentioned	1	5 × 5	Not mentioned	Severe secondary dysmenorrhea	Not mentioned	Bicornate uterus with noncommunicating horn	Histopathology	Laparoscopic removal of cyst	Recovery	Not mentioned
Kriplani A et al. (21)	16	Anterior myometrium	Not mentioned	1	4 × 5	Not mentioned	Severe secondary dysmenorrhea	Not mentioned	Degenerated myoma	Histopathology	Laparoscopic removal of cyst	Recovery	Not mentioned
Kriplani A et al. (21)	24	Right uterine wall and entering broad ligament	Not mentioned	1	4 × 4	Not mentioned	Severe secondary dysmenorrhea	Not mentioned	Bicornate uterus with noncommunicating horn	Histopathology	Laparoscopic removal of cyst	Recovery	Not mentioned
Heo SH et al. (22)	54	Anterior myometrium of the uterine fundus	US, MRI, PET	1	6 × 5 × 4.5	Regular	Pelvic pain	Normal	Ubserosal leiomyoma with secondary degeneration and possible malignant transformation	Microscopic evaluation	Abdominal hysterectomy	Recovery	No
Chun SS et al. (23)	19	Left posterior uterine fundus	MRI, laparoscopy	1	3	Not mentioned	Pelvic pain and progressive dysmenorrhea	Not mentioned	Myometrial mass	Microscopic evaluation	Laparoscopic removal of cyst	Recovery	Not mentioned
English DP et al. (24)	31	Anterior corpus of the uterus	US	1	3.5 × 2.7 × 2.4	Regular	Pelvic pain	Not mentioned	Adenomyotic cyst	Cytology	Ultrasound-guided transvaginal aspiration	Clinical improv ement	No
Branquinho MM et al. (25)	17	Right uterine myometrium	US, MRI	1	3.3 × 2.5	Regular	Dysmenorrhea	Not mentioned	Juvenile cystic adenomyoma	Clinical manifestations	Oral contraceptive pills	Relief	Laparoscopic appendectomy and a laparotomy for left ovary's haemorrhagic cyst
Jain N et al. (26)	19	Right uterine myometrium	US, MRI, laparoscopy, hysteroscopy	1	Not mentioned	Irregular	Severe dysmenorrhea and menorrhagia	Not mentioned	Uterine bicorniswith right horn hematometra	Surgery	Laparoscopic removal of cyst	Recovery	Not mentioned
Jain N et al. (26)	22	Right side of uterus below the roundligament	US, laparoscopy	1	Not mentioned	Irregular	Severe dysmenorrhea, menorrhagia and secondary infertility	Not mentioned	Broad ligament fibroid	Surgery	Laparoscopic removal of cyst	Recovery	Not mentioned
Kumakiri J et al. (27)	20	Anterior uterine myometrium	US, MRI	1	3	Not mentioned	Severe dysmenorrhea	Not mentioned	Juvenile cystic adenomyoma	Histopathology	Laparoscopic removal of cyst	Recovery	Not mentioned
Cucinella G et al. (28)	25	Posterior myometrium	US, MRI	1	4.5 × 2.4	Regular	Severe and worsening dysmenorrhea and abdominal cramping	38	Not mentioned	Histopathology	Laparoscopy	Recovery	Not mentioned
Gordts S et al. (29)	44	Uterine fundus	US, hysteroscopy	1	Not mentioned	Not mentioned	Secondary infertility	Not mentioned	Not mentioned	Histopathology	Hysteroscopic resection	Not mentioned	Hysteroscopic myomectomy
Gordts S et al. (29)	38	Isthmic level of the uterus	US, MRI	1	Not mentioned	Not mentioned	Primary subfertility	Not mentioned	Intramural cyst	Not mentioned	Hysteroscopic coagulation of the cystic wall	Not mentioned	Laparoscopic left salpingo-oophorectomy
Koukoura O et al. (30)	28	Right uterine wall	US, MRI	1	4 × 3.5	Not mentioned	Dysmenorrhoea and pelvic pain	Not mentioned	Right ovarian endometriotic cyst	Histopathology	Laparoscopic excision of the mass	Recovery	Not mentioned
Pontrelli G et al. (31)	27	Posterior uterine wall	US, MRI, hysteroscopy	1	7.5	Not mentioned	Menometrorrhagia, severe dysmenorrhea	96	Bicornuate uterus	Histopathology	Hysteroscopic lesion resection	Recovery	Not mentioned
Isik Y et al. (32)	47	Cervix	US	1	7.0 × 7.5 × 6.5	Not mentioned	Menorrhagia and pelvic pain	63	Not mentioned	Microscopic evaluation	Hysterectomy	Not mentioned	Not mentioned
Baba A et al. (33)	40	Anterior uterine wall	US, MRI	1	10	Not mentioned	Abdominal mass	96.9	Degenerative uterine myoma	Histopathology	Hysterectomy	Recovery	Not mentioned
Manta L et al. (34)	20	Anterior uterine wall	US	1	4 × 4	Regular	Chronic pelvic pain	Not mentioned	Necrobiosis of a uterine fibroid	Histopathology	Surgical excision	Not mentioned	Not mentioned
Dadhwal V et al. (35)	23	Right anterior wall near the cornual end	US, laparoscopy	1	4 × 4	Regular	Severe dysmenorrhea	Not mentioned	Adenomyotic cyst	Histopathology	Laparoscopic excision of the mass	Recovery	Not mentioned
Dadhwal V et al. (35)	16	Left uterine wall near the cornual end	US, MRI, laparoscopy	1	4 × 3	Regular	Acute episodic pain in the left lower abdomen	Not mentioned	Juvenile cystic adenomyoma	Histopathology	Laparoscopic excision of the mass	Recovery	Not mentioned
Sun W et al. (36)	47	Uterine wall	US	1	Not mentioned	Not mentioned	Menorrhagia and painful menstruation	45.8	Solitary cystic mass	Histopathology	Hysteroscopic lesion resection	Recovery	Not mentioned
Yin W et al. (37)	37	Front uterine wall and extend to the fundus and right wall	US, hysteroscopy	1	6 × 5 × 5	Regular	Heavier and prolonged menstruation as well as pelvic pain	Not mentioned	Submucous myoma	Histopathology	Hysteroscopic lesion resection	Recovery	Hysteroscopic myomectomy, abdominal myomectomy, cesarean section
Fan YY et al. (38)	36	Anterior uterine wall	US, hysteroscopy	1	4 × 3 × 4	Irregular	Increase in menstrual blood volume, dysmenorrhea	Not mentioned	Endometrial polyp or cystic adenomyosis	Histopathology	Hysteroscopic lesion resection	Recovery	A cesarean section, three induced abortions
Fan YY et al. (38)	39	Posterior uterine wall	US, MRI	1	5 × 4 × 4	Not mentioned	Relapse of uterine fibroids	1,212	Uterine fibroids with degeneration	Histopathology	Hysterectomy	Recovery	Cesarean section, hysteromyo mectomy
Li C et al. (39)	38	Right lateral wall	US, laparoscopy	1	10.4 × 5.5 × 6.0	Regular	Dysmenorrhoea	Not mentioned	Not mentioned	Histopathology	Laparoscopic excision of the mass	Recovery	Not mentioned
Zhou Y et al. (40)	45	Posterior uterine isthmus	US, MRI	1	9	Not mentioned	Abnormal uterine bleeding and progressive dysmenorrhea	8	Endometriosis cystic mass	Histopathology	Laparoscopic excision of the mass	Recovery	Laparoscopic myomectomy
Gomez NF et al. (41)	65	Posterior dome of the fundus	CT	1	7.3	Not mentioned	Pelvic mass	52.9	Gynaecologic malignancy	Histopathology	Hysterectomy	Recovery	Not mentioned
Zhou XJ et al. (42)	29	Right lateral wall	US, MRI	1	2.5 × 2.0 × 2.2	Not mentioned	Severe dysmenorrhea	Not mentioned	Cystic adenomyosis	Clinical manifestations	HIFU	Recovery	Not mentioned
Zhou XJ et al. (42)	34	Posterior wall	US, MRI	1	3.2 × 3.4 × 3.0	Not mentioned	Severe dysmenorrhea	Not mentioned	Cystic adenomyosis	Clinical manifestations	HIFU	Recovery	Not mentioned
Zhou XJ et al. (42)	20	Posterior wall	US, MRI	1	3.6 × 4.0 × 3.0	Not mentioned	Severe dysmenorrhea	Not mentioned	Cystic adenomyosis	Clinical manifestations	HIFU	Recovery	Not mentioned
Zhou XJ et al. (42)	22	Left lateral wall	US, MRI	1	2.0 × 2.0 × 2.0	Not mentioned	Severe dysmenorrhea	Not mentioned	Cystic adenomyosis	Clinical manifestations	HIFU	Recovery	Not mentioned
Jha S. (43)	28	Left cornu of the uterus	US, laparoscopy	1	3.3 × 1.2	Regular	Severe progressive dysmenorrhea	Not mentioned	Not mentioned	Histopathology	Surgical excision	Recovery	Induced abortion
Tanvir T et al. (44)	52	Posterior uterine wall	US	1	2 × 2	Regular and pain-free	Severe dysmenorrhea	Not mentioned	Not mentioned	Histopathology	Hysteroscopic resection	Recovery	Not mentioned
Arya S et al. (45)	18	Left lateral myometrium	CT, US, MRI, laparoscopy	1	3 × 3	Regular	Severe dysmenorrhea	Not mentioned	Mullerian anomaly	Histopathology	Laparoscopic excision of the mass	Recovery	Not mentioned
Arya S et al. (45)	16	Right lateral myometrium	CT, US, laparoscopy	1	5.1 × 3.6 × 4.8	Regular	Severe dysmenorrhea	Not mentioned	Mullerian anomaly	Histopathology	Laparoscopic excision of the mass	Recovery	Not mentioned
Zhao CZ et al. (46)	30	Left anterior wall	US, laparoscopy	1	5.5 × 4.0 × 5.0	Not mentioned	Severe dysmenorrhea	76.2	Not mentioned	Histopathology	Laparoscopic excision of the mass	Recovery	No

*HIFU, High intensity focused ultrasound; MRI, Magnetic resonance imaging; PET, Positron emission tomography; US, Ultrasound*.

The cause of uterine cystic adenomyosis is unclear, and researchers have proposed different theories. Uterine cystic adenomyosis is divided into two types according to the age of onset: juvenile and adult, with different etiologies. Some researchers have suggested that JCA results from developmental defects of the Mullerian ducts, leading to duplication or persistence of paramesonephric tissue (15, 47). Takeuchi et al. considered it to be a cystic variant of adenomyosis (48). The pathogenesis of the adult type is different from that of the juvenile type, and one hypothesis, accepted by most researchers, is the endometrial injury invagination theory (49). Previous uterine surgery and injury to the endometrial-myometrial junction may be the pathological basis of the disease (43). A previous history of miscarriage, parity, and curettage is associated with high risk of endometrial and myometrial injury. The resultant damage in the junction between endometrium and myometrium can cause secondary adenomyosis, which occasionally evolves into uterine cystic adenomyosis (39). Previous reports have identified eight cases of uterine cystic adenomyosis developing in the presence of the above-mentioned high-risk factors (11, 29, 37, 38, 40, 43). However, the present case had not previously undergone any surgery.

All cases of uterine cystic adenomyosis involved single lesions. At present, there are no reports of multiple lesions in the literature. The lesions can involve the uterine body, cervix, and fundus, with the uterine body and especially the right wall being the most common (41/56, 73.21% and 13/41, 31.71%, respectively), The size of the lesions varies from 2 to 20 cm, with an average diameter of ~5.5 cm. The present case was also a single lesion involving the posterior wall of the uterus, with a diameter greater than the average.

Transvaginal ultrasonography is the preferred method of examination for gynecological diseases; MRI also plays an important role in the evaluation of these cystic lesions, and was used in ~59% of cases (33/56 cases). Transvaginal ultrasonography can determine the location of the mass, distinguish between the cystic and solid components, and whether they are separated from the normal uterine cavity. The cystic part of the uterine cystic adenomyosis is mostly accompanied by dense echo spots, with moderate echoes in the cyst wall. The typical MRI findings in uterine cystic adenomyosis are a well-defined cystic lesion filled with hemorrhagic fluid in the myometrium. The liquid part of the cyst shows a hyperintense signal on both T1-weighted and T-2 weighted images, and the cystic wall shows low signal on T2 weighted images. The rim of hemosiderin in uterine cystic adenomyosis is represented by a hypointense signal on both T1-weighted and T-2 weighted images (25). The differential diagnosis includes congenital anomaly with hematometra in a Non-communicating horn, congenital uterine cysts, intramyometrial hydrosalpinx, and fibroid degeneration. In hemorrhagic hysteromyoma, methemoglobin accumulates in the periphery, producing a T1-hyperintense and T2-hypointense rim, which is different from the hypointense rim of hemosiderin in uterine cystic adenomyosis. Adipose tissue can be distinguished by a fat suppression sequence to exclude the possibility of steatosis of leiomyoma (50). Congenital uterine cysts and hydrosalpinx in the myometrium can also present as cystic lesions in the myometrium. However, the fluid composition of these lesions is simple, and the cysts lack a hemosiderin rim. MRI is also helpful in identifying complex uterine malformations (51). In cases of cystic masses of the uterine myometrium, without adequate visualization of both uterine horns on MRI or ultrasonography, it is necessary to rule out isolated congenital anomalies with hematometra in a Non-communicating horn. Hysterosalpingography may be useful, if necessary.

Elevated serum CA-125 has been proposed as a diagnostic tool for uterine cystic adenomyosis, but its specificity and sensitivity are low (38). Only 16 of the published cases reported serum CA-125 levels which were slightly increased.

It should be noted that although uterine cystic adenomyosis is a benign disease, there are three reports in the literature of malignant tumors originating from this disease (18, 33, 41). Among the three cases of cystic mass, one case had a solid component, one was nodular, and one had multiple excrescences (18, 33, 41). The patients were 40, 54, and 65 years of age, respectively. Therefore, the possibility of malignant tumors needs to be ruled out especially in older patients with solid components of cystic lesions. In addition to MRI and ultrasound, positron emission tomography (PET) can assist in the differential diagnosis by providing information on the metabolic activity of the lesion. With 18F- fluorodeoxyglucose (FDG) PET, FDG generally accumulates in malignant lesions due to high glucose metabolism. Most malignant uterine tumors, such as endometrial cancer, cervical cancer, and uterine sarcoma, usually show intense FDG uptake (52).

Inhibition of menstruation with continuous OC, gonadotropin-releasing hormone analogs, and Non-steroidal anti-inflammatory drugs may provide temporary and partial pain relief in uterine cystic adenomyosis, but symptoms may relapse after withdrawal. Because many patients with uterine cystic adenomyosis are young and wish pregnancy, minimally invasive surgery to preserve the uterus is desirable. Laparoscopic surgical resection is suitable for lesions in the myometrium, close to the serosal layer. Laparoscopic resection can significantly improve related dysmenorrhea and increase the possibility of successful pregnancy (39, 46). Robot-assisted laparoscopic management allows an optimal view and more efficient multilayer closure of the hysterotomy, thereby increasing the safety of future pregnancies (20). Hysteroscopic surgery is another minimally invasive treatment. Hysteroscopic resection of the lesion is the preferred mode of treatment for the submucosal subtype (38). However, laparoscopic or hysteroscopic surgery destroys the muscle layer surrounding the lesion in isolated cystic adenomyosis, thus increasing the risk of obstetric complications, including an increased risk of uterine rupture during pregnancy. It is also difficult to avoid the occurrence of new iatrogenic endometriosis during surgery (42). Zhou et al. reported four cases of cystic adenomyosis treated with high-intensity focused ultrasound, with resultant disappearance of dysmenorrhea and high patient satisfaction (42). Koga et al. reported a case treated with four vaginal aspirations, followed by infusions of ethanol, minocycline, and danazol without cure (11). Ryo et al. described a case treated with radiofrequency ablation with disappearance of the cystic lesion and symptomatic improvement (12).

Ethanol sclerotherapy of ovarian endometrioma has been proven to be a safe and effective minimally invasive procedure (53, 54). The pathological feature of cystic adenomyosis is endometriosis. Therefore, the successful use of ethanol sclerotherapy for ovarian endometrioma can be extended to sclerotherapy for uterine cystic adenomyosis. The reported incidence of abdominal pain during ethanol sclerotherapy for endometriotic ovarian cysts is 1.8–15.3% (55). The major ingredient of lauromacrogol is polyoxyethylene lauryl ether, in addition to ethanol and sterilized water. It is not only a type of foam sclerotherapy, but it also functions as a local anesthetic (56), which is widely used clinically. Therefore, the drug not only has a therapeutic effect, but also alleviates discomfort during treatment. Furthermore, the injection of lauromacrogol is easier and safer than the injection of absolute alcohol. Xu et al. indicated that lauromacrogol sclerotherapy is safe and effective in patients with hepatic cysts (57). A preliminary experimental study showed that lauromacrogol injection produced significant regression of endometrial foci (58). Ultrasound-guided aspiration sclerotherapy using lauromacrogol is a successful and effective treatment for refractory long-course ovarian endometrial cysts (59). In the present case, the functional endometrial tissue within the lesion was completely ablated by lauromacrogol, and the muscle layer surrounding the lesion was not damaged, retaining the integrity of the uterine wall. In previous literature the follow-up time was relatively short, and information on pregnancy and childbirth was generally missing. Therefore, we cannot judge whether the operation has an impact on the uterus during pregnancy. In our case, the long-term follow-up of our patient from procedure to pregnancy and childbirth proved that lauromacrogol is safe and effective in the treatment of uterine cystic adenomyosis. Additionally, the cost of aspiration and sclerotherapy is very low, which makes it a good choice for patients who want to reduce healthcare expenses.

Our report is limited by the fact that the cystic lesion was not surgically removed; therefore, immunohistochemical examination could not be performed. However, the combination of clinical symptoms, imaging findings and cytology are in agreement with previous literature and fully support a diagnosis of uterine cystic adenomyosis.

## Conclusion

In conclusion, uterine cystic adenomyosis is rare and can be easily misdiagnosed. Transvaginal ultrasound and MRI are of great value for diagnosis. Ultrasound-guided transvaginal aspiration and sclerotherapy for uterine cystic adenomyosis can not only effectively treat the lesion, but also preserve the integrity of the uterine wall and minimize the risk of uterine rupture during pregnancy. The present case is the first report of ultrasound-guided transvaginal aspiration and lauromacrogol sclerotherapy for uterine cystic adenomyosis with long term follow-up, and proves that it is a safe and effective minimally invasive treatment.

## Data Availability Statement

The original contributions presented in the study are included in the article/Supplementary Material, further inquiries can be directed to the corresponding author.

## Ethics Statement

Written informed consent was obtained from the participant for the publication of this case report. Ethical approval was given by the Medical Ethics Committee of our hospital.

## Author Contributions

YY diagnosed and treated the patient. All authors wrote and revised the manuscript. All authors contributed to the article and approved the submitted version.

## Conflict of Interest

The authors declare that the research was conducted in the absence of any commercial or financial relationships that could be construed as a potential conflict of interest.

## Publisher's Note

All claims expressed in this article are solely those of the authors and do not necessarily represent those of their affiliated organizations, or those of the publisher, the editors and the reviewers. Any product that may be evaluated in this article, or claim that may be made by its manufacturer, is not guaranteed or endorsed by the publisher.
