# Supplementary figures and images for: Ultrasound-Guided Transvaginal Aspiration and Sclerotherapy for Uterine Cystic Adenomyosis: Case Report and Literature Review
Source: Front Med (Lausanne). 2022 Mar 3;9:764523. doi: 10.3389/fmed.2022.764523 (PMC8928164; doi:10.3389/fmed.2022.764523)

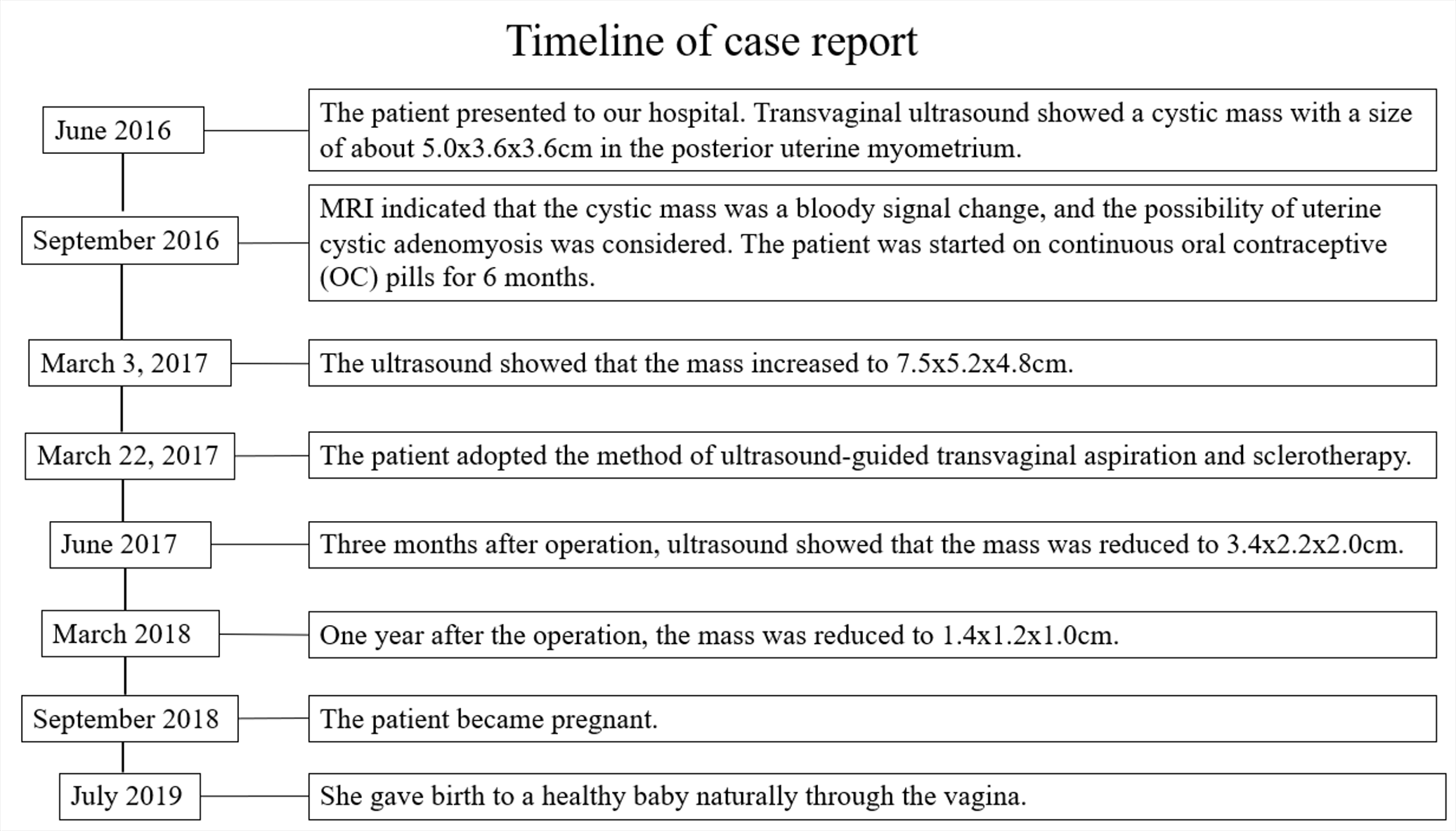

Supplement: Supplementary file 1 [file Image_1.TIF]
